# SEX- AND WORKING-STATUS SPECIFIC WORKING LIFE EXPECTANCY AMONG OLDER ADULTS IN CHILE

**DOI:** 10.1093/geroni/igae098.1419

**Published:** 2024-12-31

**Authors:** Yasuhiko Saito, Moises Sandoval

**Affiliations:** Nihon University, Tokyo, Japan; University of Chile, Santiago, Region Metropolitana, Chile

## Abstract

The mandatory retirement age in Chile, 65 years for males and 60 years for females, was set in the 1980s. However, in view of accelerated population ageing in the country, there may be a need to reconsider these age thresholds. Thus, we estimate working life expectancy, i.e., years of remaining life spent working, at older ages by sex in Chile using (working) status-based multistate life table methods. Nationally representative longitudinal data on 1313 males and 1805 females from the Social Protection Survey, conducted in 2004, 2006, 2009 and 2015, were used to estimate working life expectancy at age 65 years for males and age 60 years for females. Men working at age 65 years, on average, could expect to live 21.6 years, with working life expectancy of 2.7 years (12.5% of life expectancy). Men not working at age 65 years could expect to live 21.2 years, with a much lower working life expectancy (0.2 years; 0.9% of life expectancy). A similar pattern was observed for women – those working at age 60 years could expect to live 25.9 years, with working life expectancy of 3.0 years (11.6% of life expectancy), and those not working at age 60 could expect to live 25.8 years, with only 0.5 years of working life expectancy (1.9% of life expectancy). The findings suggest the strong influence of the mandatory retirement age on working life expectancy in Chile and its role as a policy lever to increase working life expectancy among older adults.

